# Antimicrobial, antibiofilm, and antiurease activities of green-synthesized Zn, Se, and ZnSe nanoparticles against *Streptococcus salivarius* and *Proteus mirabilis*

**DOI:** 10.1007/s00449-025-03130-8

**Published:** 2025-02-05

**Authors:** Sumeyra Gurkok, Murat Ozdal, Tuba Cakici, Esabi Basaran Kurbanoglu

**Affiliations:** 1 Department of Biology, Science Faculty, Ataturk University, 25240 Erzurum, Turkey; 2 Department of Electrical and Energy, Ispir Hamza Polat Vocational School of Higher Education, Ataturk University, Erzurum, Turkey

**Keywords:** Zn, Se, ZnSe nanoparticles, Antimicrobial, Antibiofilm, Antiurease, *Streptococcus salivarius*, *Proteus mirabilis*

## Abstract

This study assesses the antimicrobial, antibiofilm, and antiurease properties of selenium (Se), zinc (Zn), and zinc selenide (ZnSe) nanoparticles (NPs) against clinically pathogenic strains of *Streptococcus salivarius* and *Proteus mirabilis*. The Se, Zn, and ZnSe NPs, synthesized by *Pseudomonas aeruginosa* OG1, were characterized using transmission electron microscopy (TEM) revealing average sizes of approximately 30 ± 10 nm, 30 ± 15 nm, and 40 ± 10 nm, respectively. Atomic force microscopy (AFM) was used to examine the morphological and topological characteristics of the NPs. The structural and crystal characteristics were investigated using X-ray diffraction (XRD). Among the evaluated NPs, Zn NPs at a concentration of 200 mg/mL exhibited the most substantial growth inhibition zone against *S. salivarius*. Conversely, the highest antibiofilm activity was observed against *P. mirabilis*, notably with 200 µg/mL Zn NPs. In the context of antiurease activity, both 100 μg Zn and ZnSe NPs caused complete urease inhibition (100%) in *P. mirabilis* within the initial 5 h, with notable inhibition rates of 94% and 80%, respectively, observed against *S. salivarius*. Significantly, in the current landscape of NP research primarily focused on antimicrobial and antibiofilm properties, our study stands out due to its pioneering exploration of antiurease activities with these NPs. This distinctive emphasis on antiurease effects contributes original and unique value to our study, offering novel insights into the broader spectrum of NP applications, and paving the way for potential therapeutic advancements.

**Supplementary Information:**

The online version contains supplementary material available at 10.1007/s00449-025-03130-8.

## Introduction

Compared to their bulk counterparts, nanoparticles (NPs), which are relatively small materials with sizes ranging from 1 to 100 nm, offer far superior biological, chemical, and physical properties. Because of their greater surface reactivity compared to bulk materials, NPs have a variety of special characteristics [1, 2].

Despite the fact that NPs can be produced using a variety of physical and chemical techniques [3], their use in biomedical applications has been restricted because of their toxicity and bio-incompatibility [4, 5]. Conversely, biological synthesis is a cheap, safe, non-toxic, biocompatible, and environmentally acceptable green method [6, 7]. NPs can be produced by algae, plants, bacteria, fungi, viruses, and protozoa [3, 5]. However, biosynthesis with microorganisms is very popular and widely studied. The microorganisms are particularly advantageous for nanoparticle synthesis compared to other biological sources due to their scalability and efficiency. They can be cultured rapidly and in large quantities under controlled conditions, making the process cost-effective and suitable for industrial applications. Additionally, microorganisms possess diverse metabolic pathways that allow them to reduce metal ions and synthesize nanoparticles with precise size and shape control. This inherent versatility and the ability to tailor nanoparticles for specific applications make microorganisms a preferred choice for biological synthesis [8].

Intracellular or extracellular microbial NP synthesis is notably carried out by *Pseudomonas* and *Bacillus* species. These microorganisms act as significant biological nanofactories, utilizing various reductase enzymes to reduce, accumulate, and detoxify heavy metals [8]. In addition to the synthesis of nanoparticles through the reduction of ions by enzymes such as nitrate reductase, polysaccharides, biosurfactants, organic acids, pigments, and proteins also play crucial roles in the microbial synthesis of NPs [9, 10]. Furthermore, the capacity of microorganisms to grow quickly, easily and under ambient pH, temperature, and pressure settings has earned them a distinct place in the world of NP production [11–13].

In recent years, nanotechnology studies have been increasing rapidly because of their wide range of possible applications such as computer, energy, food, environment, optic, textile, cosmetics, and biomedicine [15, 16]. Because of their biocompatibility, they have many potential uses also in the field of health. In this regard, the use of NPs against health-threatening microorganisms has gained importance in recent years [14] [18, 19]. *S. salivarius* is a Gram-positive and pathogenic streptococcus [17]. It is a ureolytic and biofilm forming microorganism, present in large quantities on the oral cavity [18]. Oral biofilm production results in caries [19], whereas ureolysis induces the development of tartar or calculus [20] and raised inflammation in the gingiva and periodontal pockets [21]. Urease is also important for maintaining the stability of the biofilm consortium [22]. *P. mirabilis* is an ureolytic, Gram-negative, biofilm-forming bacterium that is frequently responsible for urinary tract infections. The creation of biofilm is a primary contributor to the onset of these infections[23]. One pivotal factor in both biofilm formation and urine crystal production by *P. mirabilis* is bacterial urease. Certain uropathogens, like *P. mirabilis*, utilize urea as a nitrogen source by expressing urease. This enzymatic activity leads to the hydrolysis of urea into ammonia and carbon dioxide, altering the local environment to become alkaline. Consequently, this process leads to the precipitation of network-forming polyvalent ions within biofilms on urinary epithelial surfaces and catheters[24]. Biofilm development and antibiotic resistance represent critical virulence factors that pose significant challenges in medical care worldwide [25, 26]. Therefore, researchers have focused on studying the effects of various NPs, including Ag, ZnO [27], Ag [28], ZnO, CuO [29], and Se [30], in an effort to inhibit the formation of biofilms and the proliferation of microorganisms.

It is also acknowledged that bacterial urease plays a significant role in the pathogenicity of potentially life-threatening situations such dental plaque, pyelonephritis, gastritis, gastric ulceration, urinary stone development, and catheter blockage [31]. NPs such as Ag, Au [32], and ZnO [33, 34] have the potential to inhibit urease. Therefore, targeting urease activity and biofilm formation using NPs may be an effective strategy for controlling pathogenic bacteria such as *S. salivarius* and *P. mirabilis*.

In the current field of NP research, which typically focuses on the antimicrobial and antibiofilm properties of NPs, our study distinguishes itself by pioneering the investigation of how these NPs affect urease activity. This unique focus adds original value to our work, providing fresh insights into the broader applications of NPs. By exploring their impact on bacterial urease activity, our research expands the understanding of NPs and opens avenues for potential therapeutic advances in treating conditions associated with urease enzyme. This innovative approach highlights the versatility of NPs and their potential to address various biomedical challenges. Therefore, in this study, the antibacterial, antibiofilm, and antiurease features of bacterially generated NPs against *S. salivarius* and *P. mirabilis* pathogens were studied.

## Materials and methods

### Microorganisms

The NPs were produced using *Pseudomonas aeruginosa* OG1 (NCBI KC453990) and their biological activities were assessed against *Proteus mirabilis* (ATCC 12453) and *Streptococcus salivarius* (ATCC 13419).

### Preparation and characterization of NPs

*P. aeruginosa* OG1 strain [6, 35] was cultured in nutrient broth (NB) media for 24 h at 30 °C and 150 rpm to synthesize the Se, Zn, and ZnSe NPs. One-hundred milliliters of Tryptic soy broth (TSB) containing 1 mM Na_2_SeO_3_, 1 mM ZnSO_4_, and 0.5 mM Na_2_SeO_3_ + 0.5 mM ZnSO_4_ was inoculated with a 500-μL cell suspension (OD_600_ = 1). The process of biosynthesis of NPs was conducted for 72 h at 30 °C and 150 rpm in the dark. Suspensions of cells were sonicated for 10 min at 100 W in an Elma/S30 ultrasonic bath to purify NPs, and then they were centrifuged. Five milliliters of hexane, ethanol, and deionized water were used to remove bacterial impurities from NPs. At each stage, centrifugation was carried out for 10 min at 10,000 g and 4 °C. The collected precipitates were then dried for 12 h at 60 °C in a hot oven. To create NPs, the obtained powders were finely mashed in a mortar and pestle [36]. The control experiments without Na_2_SeO_3_ and ZnSO_4_ were performed simultaneously.

### Characterization techniques of the NPs

Optical analyses of the Se, Zn, and ZnSe NPs were made with UV–Vis double beam spectrophotometer. The spectra were analyzed between 200 and 700 nm wavelength. The TEM (HT-7700), FE-SEM equipped with EDX (Sigma 300 Model Zeiss Gemini), Fourier transform infrared spectroscopy (FTIR Bruker Vertex), AFM (AFM500 series scanning probe microscope), and X-ray diffractometer (XRD Bruker D2, Kα, λ = 1.54 A, scanning angle 70°) were used to analyze the dimensions, forms, surface structure, and crystal configurations of Se, Zn, and ZnSe NPs and thin films [6, 37, 38].

### Antimicrobial effects

The agar disc diffusion method [39] was utilized to assess the antibacterial properties of NPs against *S. salivarius* and *P. mirabilis*. In NB, bacterial cultures were cultivated for 18 h at 150 rpm and 37 °C. One-hundred milliliter aliquots of bacterial dilutions equal to the 0.5 McFarland standard (1.5 × 108 cfu/mL) were spread out on nutrient agar (NA) plates. These plates were covered with paper discs impregnated with 100 μg and 200 μg of the test NPs, a positive control disc containing 25 μg of gentamicin, and a negative control disc containing 25 μL of sterile ddH_2_O. The antibacterial activities of the NPs were evaluated by measuring the inhibition zones surrounding the control and NPs-impregnated disks after they were incubated for 24 h at 37 °C [37].

### Antibiofilm effects

In 96-well culture plates, the biofilm inhibition experiment was conducted using Zn, Se, and ZnSe NPs against *S. salivarius* and *P. mirabilis*. Following a 24-h incubation period at 37 °C in 10 mL of TSB containing 1% glucose, dilutions equal to the 0.5 McFarland standard were obtained. Ten microliters of test bacterial dilutions, 12.5 µL of test NPs (final concentrations of 100 µg/mL and 200 µg/mL), and 177.5 µL of growth media were combined in each well of the plates. While the negative control contained simply growth media, the positive controls included 190 µL of growth medium mixed with 10 µL of the bacterial dilutions. After the 24-h incubation period at 37 °C, the 96-well plate was rinsed three times with distilled water to eliminate any loose planktonic cells. The adherent sessile cells that remained were then stained for 30 min using 200 μL of 0.4% crystal violet. The excess dye was then poured out, and the wells were rinsed three times with distilled water. After 30 min at room temperature, the leftover colored biofilm was suspended in 200 µL of 70% ethanol. Using a microplate reader (Thermo Scientific Inc., Multiskan GO, Finland), the wells’ OD was measured at 570 nm [40]. The following formula was used to determine biofilm inhibition:

$${\text{Biofilm inhibition }}\left( \% \right) \, = \, \left[ {\left( {{\text{Control OD}}_{{{57}0{\text{nm}}}} {-}{\text{ NP}} - {\text{treated OD}}_{{{57}0{\text{nm}}}} } \right)/{\text{ Control OD}}_{{{57}0{\text{nm}}}} } \right] \times {1}00$$

### Urease inhibition assay

Spectrophotometric measurement at 425 nm was used to evaluate urease activity using the Nesslerization method [41]. After incubating the test strains *S. salivarius* and *P. mirabilis* in NB for 24 h at 37 °C, suspensions equal to the 0.5 McFarland standard were made. The urease assay was carried out in test tubes holding 200 μL of urea solution (0.6 M urea was made in tris buffer, pH 7) and 3.8 mL of NB. Thirty μL of test bacterial suspensions (0.5 McFarland) were added together with test NPs (final concentration of 100 μg/mL). The test NPs were not added to the positive control tubes, which were made in the same manner. The sole ingredients in the negative controls were urea and NB in the same amounts. Each tube was incubated for 5 and 24 h at 37 °C and 150 rpm. The tubes were subsequently centrifuged at 6000 rpm. The 900 μL urea solution was combined with the supernatant (100 μL) and incubated for 15 min at 30 °C. After adding 100 µL of 10% trichloroacetic acid (TCA) to end the urease reaction, the mixture was centrifuged at 6000 rpm. A mixture of 200 μL Nessler Reagent, 1.6 mL ddH_2_O, and 200 μL supernatant was incubated for 5 min at room temperature to assess the ammonia that was emitted. The UV–Visible spectrophotometer was used to measure the mixes' optical density (OD) at 425 nm. The following formula was used to determine the rates of urease inhibition:

$${\text{Urease inhibition }}\left( \% \right)\, = \,\left[ {\left( {{\text{Control OD}}_{{{\text{425nm}}}} {-}{\text{NP}} - {\text{treated OD}}_{{{\text{425nm}}}} } \right) \, /{\text{ Control OD}}_{{{\text{425nm}}}} } \right]\, \times \,{1}00$$

### Statistical analysis

Each experiment was run in triplicate. To statistically analyze the data, IBM SPSS software 22.0 (Chicago, IL, USA) was utilized.

## Results and discussion

### Optical, structural, and morphological properties of green synthesized Se, Zn, and ZnSe NPs

**Fig. 1:**
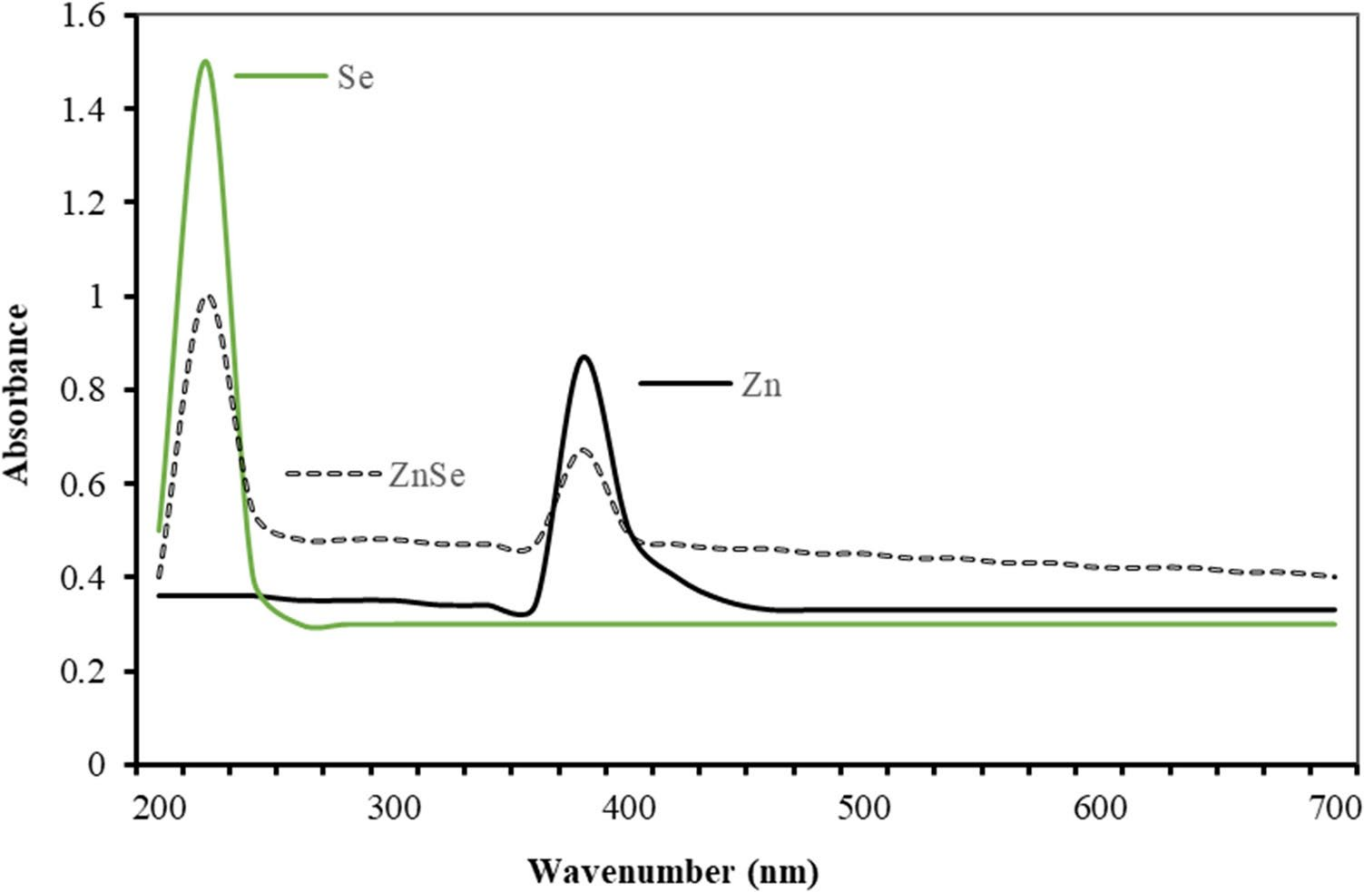
UV–Vis absorption spectroscopy of NPs

Zn and Se ions were reduced simultaneously to create Zn–Se NPs, as demonstrated by UV–visible spectroscopy. The absorbance spectrum plots of line, dot, and dot-dashed line for Se, Zn, and ZnSe NPs that were recorded in the 200–700 nm wavelength range are displayed in Fig. 1. The transmission data were used to calculate the optical band gap and absorption coefficient. The following equation was used to get the absorption coefficient (ɑ).

1 $$I = I_{0} \mathop e\nolimits^{ - ad}$$

where d is the film’s thickness and I_0_ and I are, respectively, the incident and transmitted radiation intensities. The following equation was used to get the optical band gap (E_g_) values:

2 $$ah\upsilon \, = \,A \, \left( {h\upsilon - E_{g} } \right)^{n}$$

Similarly, the band gap of NPs reduced in electron volts (eV) can be estimated on the wavelength vs absorption plot, corresponding to an approximate value between the minimum value and the maximum value of the absorption plot. At approximately 360–380 nm and 200–220 nm, the absorption maxima for Zn and Se NPs were noted, respectively (Fig. 1). According to research, ZnO NPs UV–Vis absorption peaks at 360 nm [42], 380 nm [29], and 359 nm [27] are produced during biosynthesis. The slight difference in absorption may be attributable to variations in the content and circumstances of the reaction mixture. The absorption peaks of Se NPs were identified at 297 nm [43] and 265 nm [44], respectively, in earlier investigations. Differing absorption min and max values from the literature are a result of variations in NP reduction techniques as well as defect mechanisms brought on by oxygen binding contaminations. The lower size of the Se NPs accounts for the peak at 220 nm. Single absorption peak data were acquired in the wavelength absorption graph for Zn and Se NPs reduced by the bacterial approach. However, it is seen that the ZnSe NPs reduced as a compound peak at two absorption centers (Fig. 1 dot-dashed line).

**Fig. 2:**
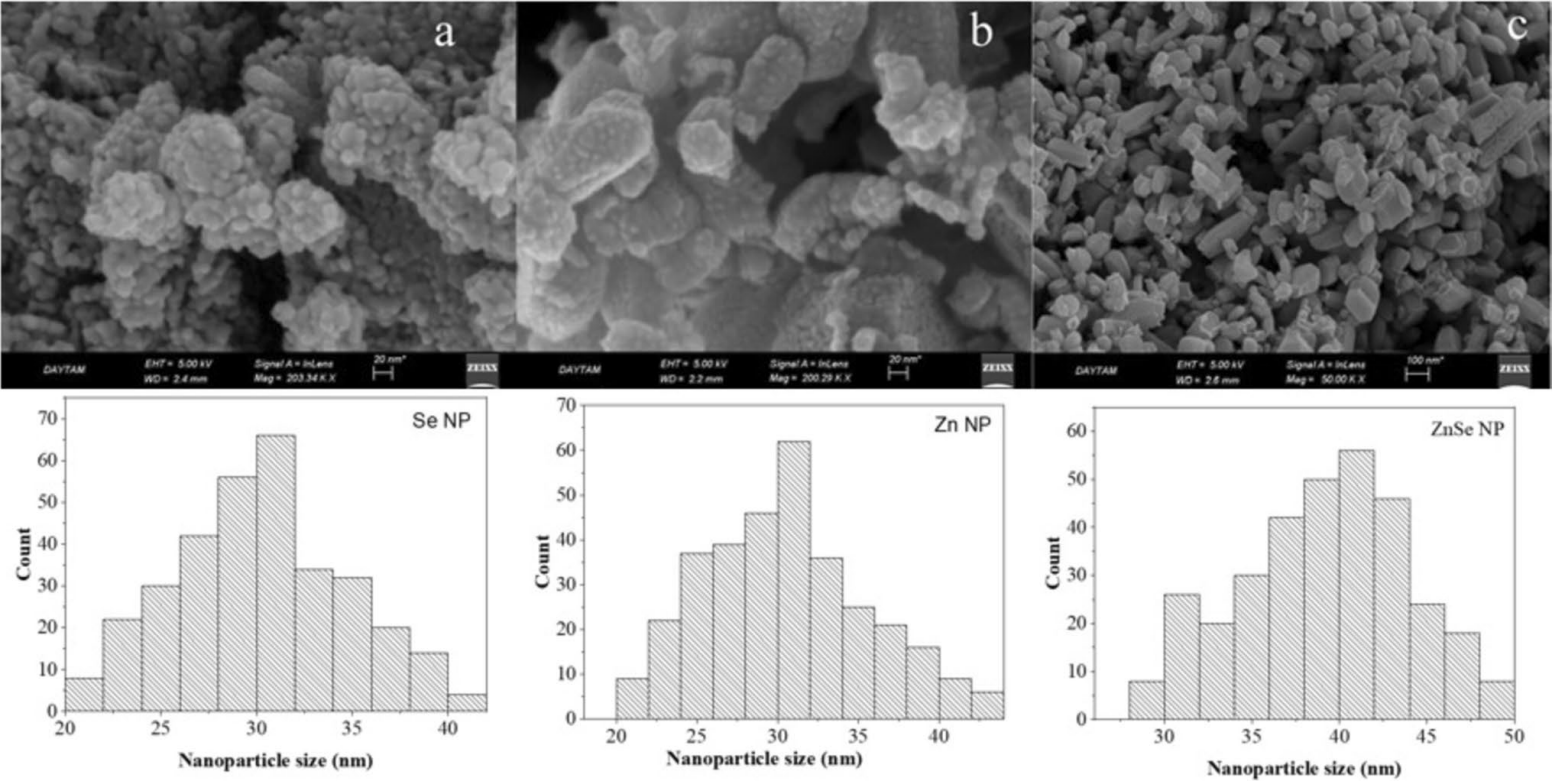
FE-SEM images and size distribution histogram of Se (**a**), Zn (**b**) and ZnSe (**c**) NPs synthesized by *P. aeruginosa*

**Fig. 3:**
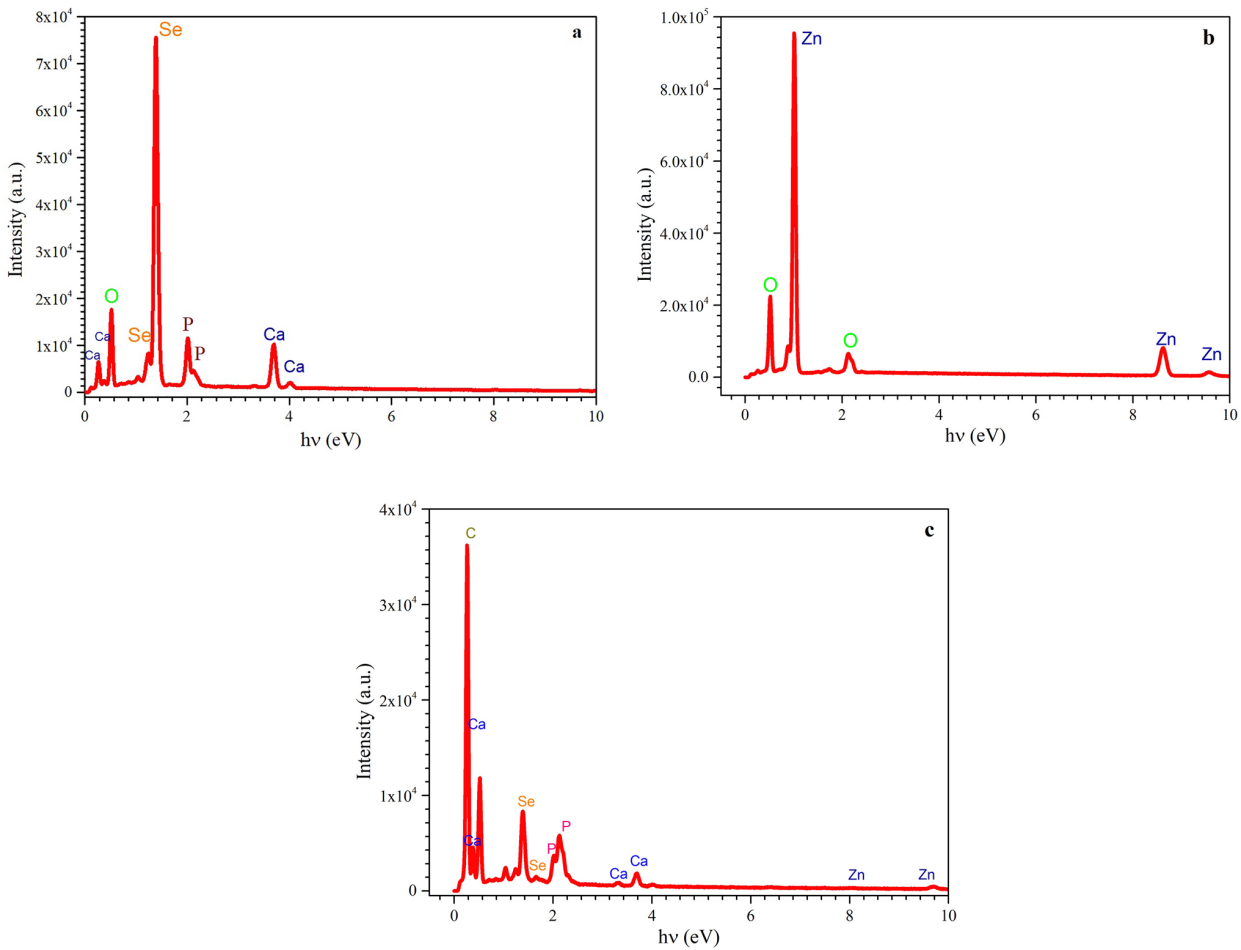
EDX results of Se (**a**), Zn (**b**) and ZnSe (**c**) NPs synthesized by* P. aeruginosa*

An essential application of the FE-SEM lies in acquiring comprehensive insights into the material composition. To conduct compositional analysis on NPs, the FE-SEM is outfitted with an EDX analysis system. To investigate the stoichiometry of NPs, a quantitative examination of the NPs is conducted utilizing the EDX approach. As seen in Figs. 2 and 3, the structural characteristics and chemical composition of the produced NPs were examined using a SEM equipped with an EDX. Figure 2 (a), (b), and (c) display sample images of Se, Zn, and ZnSe NPs obtained using FE-SEM. The microsphere-like morphology of Se NPs with a diameter of 30 ± 10 nm is depicted in Fig. 2a. In addition to their spherical form, Zn NPs exhibit a rod-like 30 ± 15 nm structure (Fig. 2b). Agglomeration was seen in certain areas as a result of the NPs’ surface action, as shown in both micrographs. ZnSe NPs are made up of 40 ± 10 nm rod-like sheeted structures, as seen in Fig. 2(c). This micrograph makes it evident that the highly organized and nearly uniform nanorod-like sheeted formations.

According to the usual EDX spectrum displayed in Fig. 3(a), (b), and (c), common elements were found in the Zn, ZnSe, and Se NPs, respectively. Strong Se and Zn signals from the EDX spectrum in Fig. 3(a) and (b), respectively, indicated the presence of relevant NPs in the biosynthesized material.

The EDX spectrum in Fig. 3(c) displays the percentage of the constituent elements derived from the ZnSe NPs EDX measurement findings. The elements analysis of Fig. 3(c) reveals the presence of elements Zn and Se. A minor proportion of Ca, O, and P components are also present. The molecules that were present in the growth media of the microorganisms are responsible for the additional Ca, O, and P peaks that were seen.

**Fig. 4:**
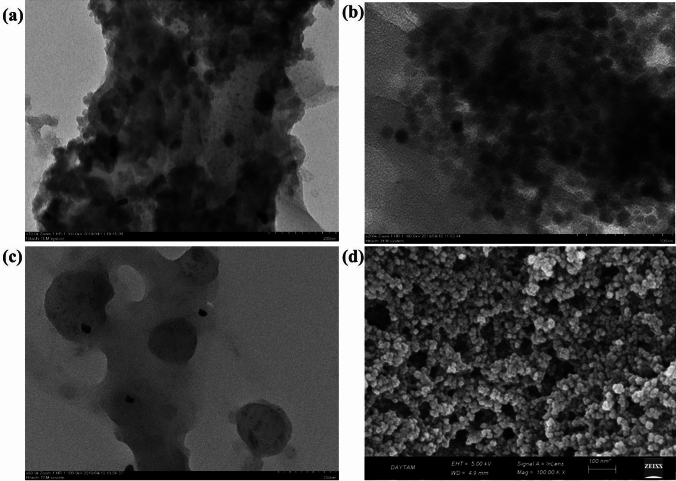
TEM images of Se, 200 nm (**a**); Zn, 100 nm (**b**); ZnSe, 200 nm (**c**) and FE- SEM image of ZnSe thin film, 200 nm

TEM images taken at the scale of 200 nm, 100 nm, and 200 nm of the NPs of Se, Zn, and ZnSe NPs are shown in Fig. 4 (a), (b), and (c), respectively. Se NPs in Fig. 4 (a) have a size of approximately 30 ± 10 nm and they are clearly seen in the hexagonal sphere form, which was confirmed by Jiang [45]. Zn NPs in Fig. 4(b) are scaled at around 30 ± 15 nm. The particle size of ZnSe NPs, which are reduced as a compound, was around 40 ± 10 nm as seen in Fig. 4(c). TEM micrographs confirm the FE-SEM results of Se, Zn and ZnSe NPs. The ZnSe nano-thin film image in Fig. 4(d), obtained using a FE-SEM on a glass substrate, reveals that the particles are all nanospheres, each measuring around 30 ± 5 nm in diameter. This allows us to examine the interior structure of the smooth nanospheres.

**Fig. 5:**
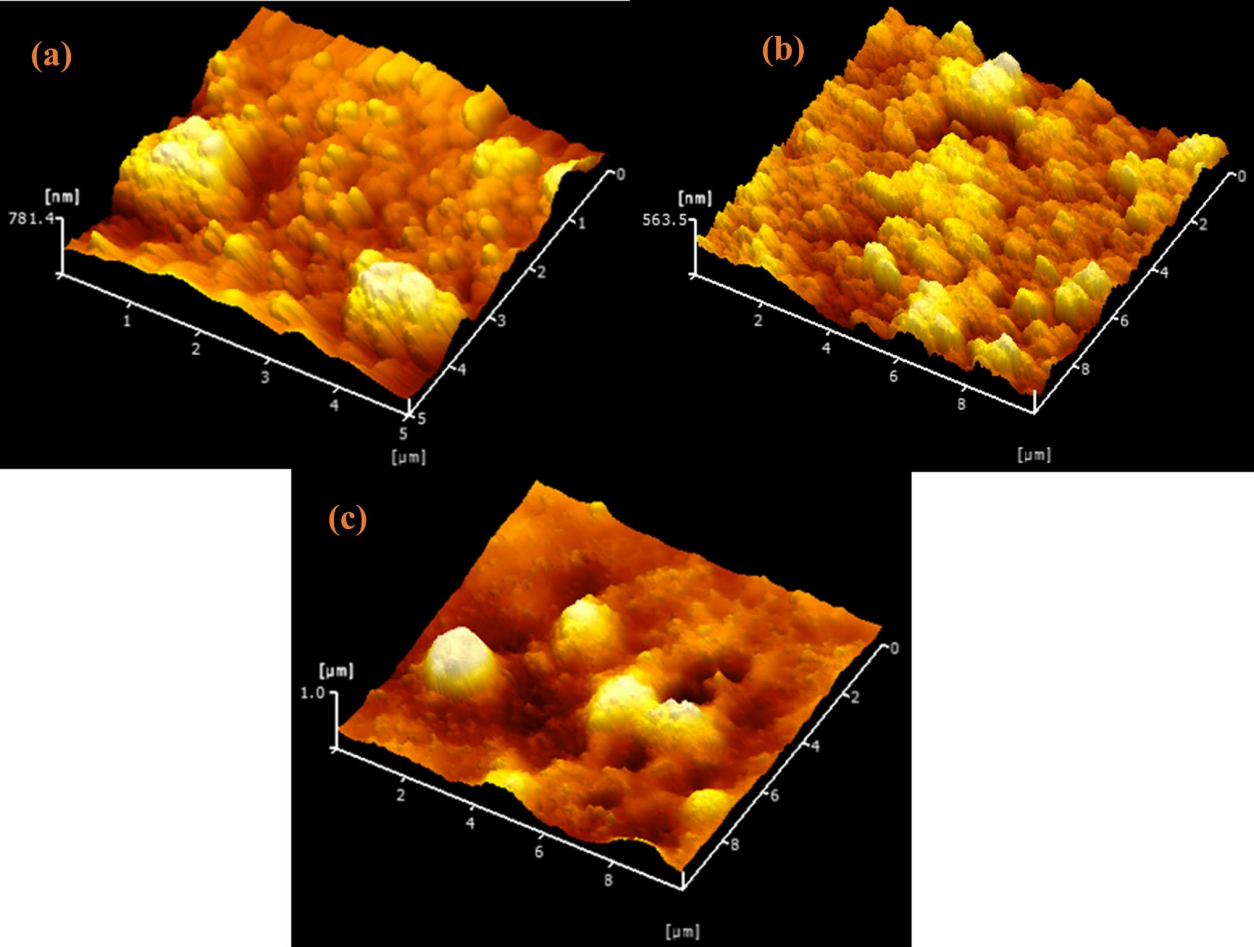
3D AFM images of Se (**a**); Zn (**b**), and ZnSe (c) NPs thin films

Three-dimensional AFM micrographs are used in Fig. 5 to illustrate the positioning and dispersion of NPs on the glass surface. Surface roughness has a significant role in charge transfer on thin films. The topography and the surface roughness of the Se, Zn, and ZnSe NPs formed a thin film are shown in Fig. 5(a), (b), and (c), respectively. The surface roughness values of Se, Zn, and ZnSe thin films were 84.44 nm, 53.88 nm, and 31.20 nm, respectively. The surface roughness of ZnSe was smaller than that of the Se and Zn thin films. The crystal phase configurations of Se, Zn, and ZnSe NPs determined from XRD data were cubic for Zn and ZnSe, and hexagonal for Se. The surface roughness value was greater in the hexagonal configuration, as assessed by AFM images.

**Fig. 6:**
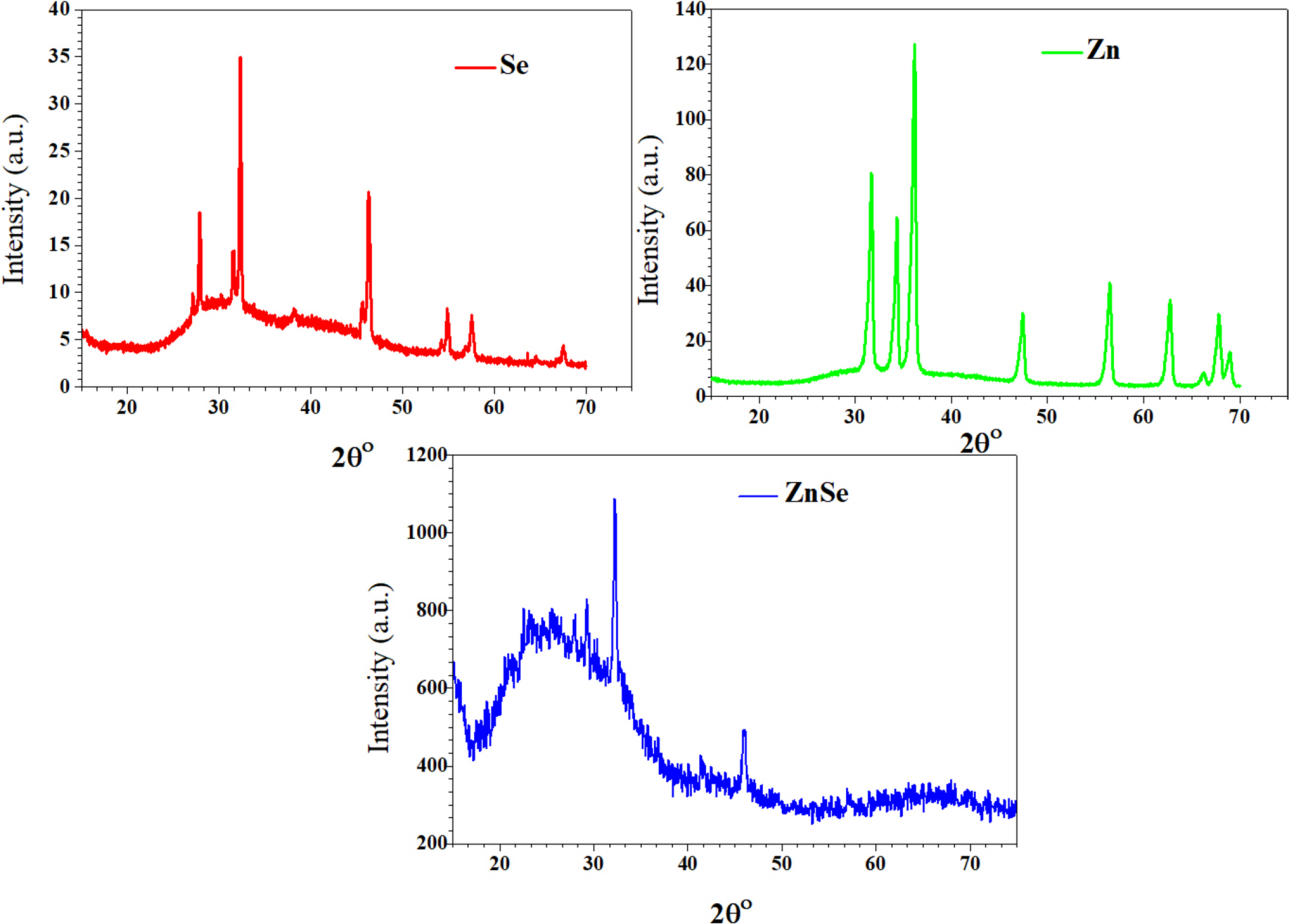
XRD patterns of the Se, Zn, and ZnSe thin films

All of the thin-film materials’ phases and crystal structures were investigated using X-ray powder diffraction. The thin films of Se, Zn, and ZnSe NPs performed structural investigation at scanning angles ranging from 10° to 80°. The red, green, and blue lines in Fig. 6 represent the XRD patterns of the Zn, Se, and ZnSe NPs, respectively. Figure 6 displays the (h,k,l) diffraction planes of the XRD peaks of the Zn, Se, and ZnSe thin films. It was determined that all thin films were polycrystalline. ZnSe thin-film XRD peaks at 2θ angles are found and they closely align with patterns that represent the cubic crystal phases of the material (PDF Nos. 01–080-0021, 01–076–1363, and 00–037–1463) [46]. The dimension of the diffraction peak can also be used to estimate the average crystallite size (D) in the film. Using the Bragg equation, the inter-planar spacing d value may be determined from the X-ray diffraction profiles. The (h,k,l) diffraction planes of the Se, Zn, and ZnSe thin films that matched the XRD peaks are also labeled in Tables 1, 2, and 3, respectively. The crystallite Se, Zn, and ZnSe thin films were computed based on the 2ʟ values of the peaks, and the results are displayed in Tables 1, 2, and 3, respectively, for inter-planar spacing and crystallite Se. These structural characteristics of the Zn, ZnSe, and Se thin films on the glass substrate are displayed in Tables 1, 2, and 3. The Scherrer equation is utilized to compute the sizes of the crystallites.

3 $$D = \frac{0,9\lambda }{{\beta \cos \theta }}$$

where Ɵ is the matching Bragg’s angle, β is the full wide at the half-maximum (FWHM) of the spectral peak, and λ is the wavelength (λ = 1.5405 Å).

**Table 1:** XRD peaks angle and diffraction planes (h,k,l) of Se thin film

(hkl)	FWHM	FWHM [rad]	Intensity [a.u]	Observed, 2θ^o^ [Deg.]	d-values [nm]	Crystal size, D [nm]	CrystalPhase
(111)	0.1624	0.00283	210.23	28.77	3,10	50.59	Cubic
(200)	0.1496	0.00261	596.91	31.99	2.79	38.17	Cubic
(201)	0.3247	0.00566	2770.64	40.92	2.20	26.15	Cubic
**(220)**	0.3300	0.00576	5342.04	45.71	1.98	26.12	Cubic

**Table 2:** XRD peaks angle and diffraction planes (h,k,l) of Zn thin film (*ZnO thin film)

(hkl)	FWHM	FWHM [rad]	Intensity [a.u]	Observed, 2θ^o^ [Deg.]	d-values [nm]	Crystal size, D [nm]	CrystalPhase
(*)	0.1968	0.00343	6016.86	31.7219	2.8191	42.03	Hegzegonal
(*)	0.2165	0.00378	4866.30	34.3535	2.609	38.40	Hegzegonal
(002)	0.2558	0.00446	10,446.87	36.1861	2.4805	32.71	Cubic/Hegzegonal
(*)	0.2362	0.00412	2066.50	47.5074	1.9127	36.78	Hegzegonal
(102)	0.1968	0.00343	3255.41	56.5265	1.6271	45.91	Cubic/Hegzegonal
(*)	0.2165	0.00378	2649.39	62.8173	1.4785	42.99	Hegzegonal
(*)	0.3149	0.00549	389.15	66.2599	1.4098	30. 17	Hegzegonal
(*)	0.0960	0.00167	2341.27	67.7923	1.3815	100.05	Hegzegonal
(112)	0.0984	0.00172	2270.45	67.8835	1.3598	97.90	Cubic/Hegzegonal
(103)	0.1680	0.00293	1074.03	69.0210	1.4098	56.52	Cubic/Hegzegonal

**Table 3:** XRD peaks angle and diffraction planes (h,k,l) of ZnSe thin film

(hkl)	FWHM	FWHM [rad]	Intensity [a.u]	Observed, 2θ^o^ [Deg.]	d-values [nm]	Crystal size, D [nm]	CrystalPhase
(111)	0.1624	0.00283	154.76	29.28	3.05	506.5001	Cubic
**(200)**	0.2165	0.00378	486.49	32.27	2.77	381.929	Cubic
**(220)**	0.3300	0.00576	156.47	46.04	1.97	261.588	Cubic

Table 2 displays the recognition and good matching of all XRD planes and 2Ɵ angles with a pattern that corresponds to a ZnO hexagonal crystal phase (PDF No: 01–079–0205; 00–003–0752; 00–023–1497) [47]. As seen from the TEM and FE-SEM structural analysis results, it can be concluded that the rod-like NPs, especially in the Zn NP structure, originate from the ZnO structure. Thus, it turns out that TEM, FE-SEM, and XRD analyses support each other to a large extent.

**Fig. 7:**
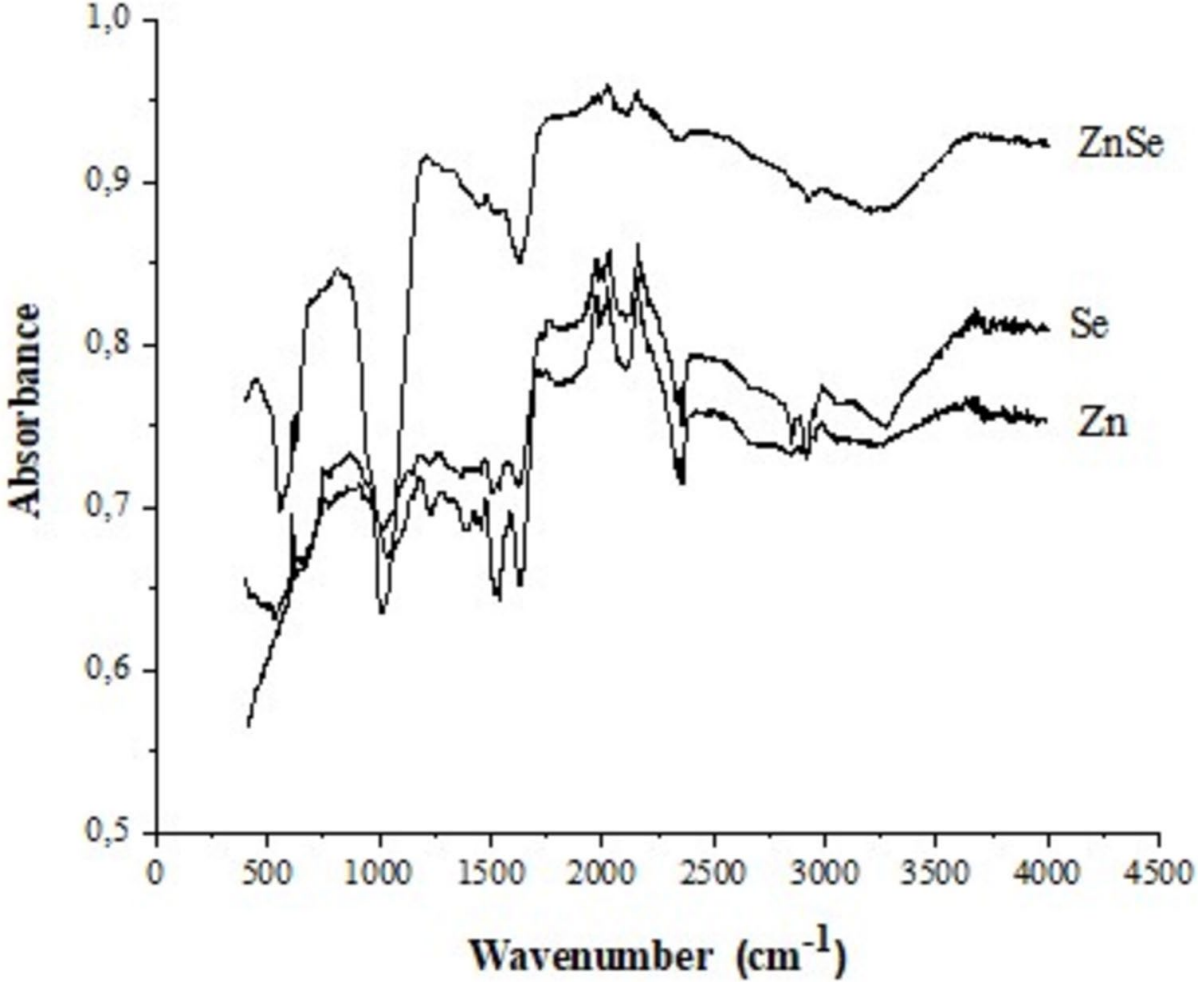
The FTIR spectrum of Zn, Se, and ZnSe NPS

*P. aeruginosa* bioactive ingredients adhere to the NPs’ surface to form a capping layer. An FTIR measurement was carried out to find out more about the biological components that are present in the structure of the NP. Significant similarities between the samples were found by FTIR examination of the NPs. Figure 7 shows that the FTIR spectra of all three NPs showed the following patterns: 2354 cm^−1^ (C = C conjugated), 2103 cm^−1^ (C≡C), 1640 cm^−1^ (C = C group), and 1038 cm^−1^ (O–H stretch). Proteins and rhamnolipids are represented by the absorption bands seen at 1046 cm^−1^ and 1020–1151 cm^−1^, respectively [48]. The FTIR peaks of the nanoparticles suggest that rhamnolipid molecules were involved in the reduction of Na_2_SeO_3_ and ZnSO_4_ to SeNPs, ZnSe NPs, and ZnNPs. It is unclear exactly how bacteria make NPs. Based on existing studies, adding metal salts to microbial media causes the metal ions to form fast bonds with proteins and water-soluble molecules containing functional groups (such -OH and -COOH) that cause the metals to become persistent [38]. As a result, the hydrophobic residues of proteins have changed in shape and are now accessible to aqueous phases. This facilitates the entry of reducing agents from culture media and encourages the conversion of trapped metals into metal NPs [38, 49].

### Antimicrobial effects of green-synthesized Se, Zn, and ZnSe NPs

To assess the antibacterial efficacy of Se, Zn, and ZnSe NPs against *S. salivarius* and *P. mirabilis*, the dimensions of the growth inhibition zones were measured. The dimensions of the zones are provided in Table 4 and the images of the petri plates are presented in Figure S1. Depending on the test strains and the type and concentration of NPs, the antibacterial effects varied. Among the NPs, the largest inhibition zones against both strains were observed with Zn NPs (200 μg/disc**)**, with the larger in *S. salivarius* strain (15 mm ± 1.8). The lowest zone (7 mm ± 1) was measured against *P. mirabilis* when Se NP (100 μg/disc) was used. Wider inhibition zones were obtained with increasing NP concentrations in both bacteria. In the positive control gentamicin discs, zone of inhibition of 18 ± 3 and 17 ± 2 mm was noted in *S. salivarius* and *P. mirabilis*, respectively (Figure S1).

**Table 4:** The diameters of the zones of grow inhibition formed by Zn, Se, and ZnSe NPs against *S. salivarius* and *P. mirabilis*

	Zone of Inhibition (mm)			
	*S. salivarius*		*P. mirabilis*	
Nanoparticles	100 μg/disc NP	200 μg/disc NP	100 μg/disc NP	200 μg/disc NP
Zn	13 ± 1.5	15 ± 1.8	10 ± 1.2	12 ± 1.2
Se	8.0 ± 1.0	11 ± 1.3	7.0 ± 1.0	9.0 ± 1.0
ZnSe	10 ± 1.3	14 ± 1.6	8.0 ± 1.0	11 ± 1.2
Gentamicin (25 μg)	18 ± 3.0	18 ± 3.0	17 ± 2.0	17 ± 2.0

ZnNP showed higher antibacterial activity than SeNP and ZnSeNP. One possible explanation is that the observed decrease in antimicrobial activity of ZnSe nanocomposite compared to Zn nanoparticles alone may be due to differences in surface properties and bioavailability. Also, ZnNPs may be more prone to oxidation than SeNP. Similar to this study, CuNPs were found to have more antimicrobial activity than Ag/CuNP and this is because CuNPs are more prone to oxidation than AgNP when positioned close to the bacterial cell membrane [50].

The generation of reactive oxygen species, disruption of the structure of the cell wall and membrane, suppression of DNA replication, and inhibition of protein synthesis are only a few of the several processes that underpin the antibacterial action of NPs [26, 30, 51]. The antimicrobial activity of Se NPs [43, 52] and Zn [27, 29] has been previously reported. Because of the varied NPs’ characteristics (size, impurity, and form), the NPs’ production process, and the examined bacteria, there are some slight variations between the findings of this investigation and those published in the literature. Many investigations have demonstrated that NPs are more efficient against Gram-negative bacteria. Manzoor [53] investigated the antibacterial efficacy of platinum NPs against four pathogenic bacteria: Gram-positive *B. cereus*, and Gram-negative *P. aeruginosa*, *K. pneumonia*, and *E. coli*. Compared to Gram-positive bacteria, platinum NPs have been shown to significantly inhibit Gram-negative bacteria. The variations in cell wall constructions are the reason for these discrepancies. The peptidoglycan layer of Gram-positive bacteria is thick, while the lipopolysaccharide-containing outer membrane (LPS) of Gram-negative bacteria is thin. The effectiveness of NPs may differ between these two bacteria groups [54, 55]. In this investigation, it was found that NPs were more efficient against Gram-positive *S. salivarius*. Similar to this, ZnO NPs were found to have a greater antibacterial effect on Gram-positive bacteria (such as *Staphylococcus aureus*) than on Gram-negative bacteria (such as *Escherichia coli* and *P. aeruginosa*) [56].

### Antibiofilm effects of green-synthesized Zn, Se, and ZnSe NPs

Antibiofilm agents are important in preventing bacterial infections and reducing resistance to antibiotics [57, 58]. To evaluate the ability of NPs to inhibit biofilm formation, the bacteria were examined after a 24-h incubation period. Biofilm formation was dependent on NP concentrations and biofilm formation was inhibited more in *P. mirabilis* than in *S. salivarius*. Biofilm inhibition was observed in both bacteria, more with Zn NP and least with Se NP. Based on the results, it was concluded that especially Zn NP and ZnSe NPs suppressed biofilm formation in both bacteria more efficiently in comparison to the SeNPs (*p* < 0.05). (Table 5). Se NPs at a concentration of 100 µg/mL inhibited *P. mirabilis* and *S. salivarius* biofilm formation by 51.4% and 40.2%, respectively. The maximum biofilm inhibition for *P. mirabilis* was observed with ZnSe NPs at a concentration of 200 µg/mL, whereas for *S. salivarius*, it was achieved with Zn NPs at a concentration of 200 µg/mL (Figure S2).

**Table 5:** Biofilm inhibition properties of Zn, Se, and ZnSe nanoparticles against *S. salivarius* and *P. mirabilis*

	Biofilm Inhibition Rate (%)			
	*S. salivarius*		*P. mirabilis*	
Nanoparticles	100 μg/ml NP	200 μg/ml NP	100 μg/ml NP	200 μg/ml NP
Zn	69.3 ± 3.1^a^	80.4 ± 3.7^a^	70.8 ± 3.4^b^	83.3 ± 4.0^b^
Se	40.2 ± 2.2^c^	60.4 ± 3.0^c^	51.4 ± 2.7^c^	62.0 ± 3.6^c^
ZnSe	60.6 ± 3.0^b^	72.6 ± 3.4^b^	82.9 ± 3.9^a^	90.3 ± 4.2^a^

Letters indicate statistically significant differences (*p* < 0.05) for each column. ± standard deviation (SD) is the unit of calculation for errors

The findings of the present study differ from some previous studies, potentially due to variations in NP synthesis methods (e.g., size, shape, surface charge, or functionalization), the bacterial strains used, or experimental conditions such as incubation times, nutrient media, or biofilm quantification techniques. According to Obeizi et al., at a concentration of 100 μg/mL of ZnO NPs, biofilm formation was inhibited by 87% in *P. aeruginosa* and 91% in *S. aureus* [42]. However, Mohamed et al. reported that ZnO NPs (10–3000 ug/mL) did not show a significant effect on *S. aureus* and *P. aeruginosa* biofilm formation [29]. It was shown by Shakibaie et al. [59] that Se NPs inhibit the biofilm formation of *P. aeruginosa*, *S. aureus*, and *P. mirabilis*. ZnSe NPs were found to suppress *P. aeruginosa* and *B. subtilis* biofilms, as reported by Mirzaei [60].

Numerous infectious illnesses, including atherosclerosis, wound infections, cystic fibrosis, urinary tract infections, mastitis, endometritis, middle ear infections, endocarditis, sialolithiasis, bacterial vaginosis, and prostatitis, are caused by biofilms [61]. Since NPs are smaller than 350 nm and have a stronger contact with negatively charged biofilm structures, their antibacterial qualities and biocompatibility make them ideal for antibiofilm applications [26, 62, 63]. NPs may limit biofilm formation through bactericidal actions, suppression of bacterial adherence to the surface, and production of oxidative stress [60, 63].

Surprisingly, ZnSe NPs caused more biofilm inhibition in *P. mirabilis* than Se NPs (Table 5). Because of their synergistic interactions, bimetallic NPs have been shown to have strong antibacterial activity against a wide range of microbes, with the majority of them outperforming their monometallic counterparts. The reason for this is that every type of metal has the ability to contribute due to its distinct physicochemical characteristics, ability to interact with other NPs and bacterial cells, and various modes of antimicrobial action [51]. This result is particularly surprising because monometallic Se NPs are known for their significant antibiofilm activity. The enhanced activity of ZnSe NPs may be attributed to the additional contribution of Zn ions, which can interfere with bacterial metabolism and biofilm integrity. Zn ions are known to destabilize bacterial cell membranes and disrupt quorum sensing, a critical process in biofilm formation. Furthermore, the presence of Zn in the ZnSe NPs might enhance the generation of reactive oxygen species (ROS), which can cause oxidative damage to bacterial cells and biofilms [64]. The combination of these effects likely explains the superior performance of ZnSe NPs compared to Se NPs alone. However, further studies investigating ROS generation and ion release dynamics are necessary to confirm these hypotheses and to better understand the mechanisms involved.

### Inhibitory effect of NPs on urease synthesis in *S. salivarius* and *P. mirabilis*

The inhibition rates of Zn, Se, and ZnSe NPs on urease synthesis, which is accepted as a virulence factor of clinical pathogens *S. salivarius* and *P. mirabilis*, are given in Table 6. NPs showed significant differences in inhibition of urase synthesis (*p* < 0.05). After 5 h of incubation with Zn and ZnSe NPs, a complete inhibition (100%) of urease activity was observed in *P. mirabilis*. In contrast, *S. salivarius* exhibited urease inhibition rates of 94% with Zn NPs and 80% with ZnSe NPs after the same incubation period. Their inhibitory effects on urease synthesis against both pathogenic bacteria were drastically reduced or completely lost after 24 h of incubation. This may be due to the growth of bacteria since the NP concentration was used below the lethal dose.

**Table 6:** Urease inhibition rates of Zn, Se, and ZnSe nanoparticles at 100 μg concentration against *S. salivarius* and *P. mirabilis* after 5 h and 24 h incubation

	Urease Inhibition Rate (%)			
	*S. salivarius*		*P. mirabilis*	
Nanoparticles	5 h	24 h	5 h	24 h
Zn	94 ± 4.2^a^	28 ± 1.2^a^	100 ± 0^a^	0 ± 0^b^
Se	70 ± 3.2^c^	15 ± 0.7^b^	80 ± 3.2^b^	0 ± 0^b^
ZnSe	80 ± 3.5^b^	12 ± 0.5^c^	100 ± 0^a^	7 ± 0.1^a^

Letters indicate statistically significant differences (*p* < 0.05) for each column. ± standard deviation (SD) is the unit of calculation for errors

Enzymes are one of the major bacterial virulence factors and the inhibition of enzymes is a simple approach to combat certain infections. NPs change the structure of the enzyme by interacting with its allosteric or active sites. Because of its non-competitive approach to NP binding, urease loses its enzymatic properties [65]. While urease inhibition activities of NPs such as Ag [66, 67], Au [67], SiO_2_, TiO_2_, ZnO [68] have been previously reported, the urease inhibitory effect of Se and ZnSe NPs has not been studied before. Therefore, these NPs represent novel candidates for future biomedical product development as urease inhibitors.

In addition to antibacterial and antibiofilm activities, this distinctive emphasis on antiurease effects contributes original and unique value to our study, offering novel insights into the broader spectrum of NP applications and paving the way for potential therapeutic advancements. Unlike conventional NP studies that predominantly focus on antimicrobial and antibiofilm properties, the present study is one of the few that investigates antiurease activities, addressing a relatively underexplored aspect of nanoparticle research. This study highlights the enhanced biofilm inhibition observed with ZnSe NPs, which may be attributed to their bimetallic nature, facilitating synergistic interactions and potentially increased ROS generation. Furthermore, the investigation of selenium (Se), zinc (Zn), and zinc selenide (ZnSe) nanoparticles against the clinically significant pathogens *Streptococcus salivarius* and *Proteus mirabilis* adds to the novelty of this research, as both the selected pathogens and NPs are less commonly studied in combination, particularly for antiurease activity. These findings not only expand the potential applications of biogenic NPs but also reinforce their promise as versatile agents in addressing multifaceted microbial challenges.

## Conclusion

This study presents biogenic NPs as an alternative antibacterial, antibiofilm, and antiurease agents that can be used against different pathogens by focusing on *S. salivarius* and *P. mirabilis* pathogens. This research highlights the significant potential of green manufactured NPs in the food, pharmaceutical, and cosmetics industries. However, before their usage in these fields that directly impact human health may be approved, more research is required to ascertain the cytotoxic effects.

In addition, a comprehensive investigation into factors such as nanoparticle synthesis methods and experimental conditions is essential to elucidate the mechanisms underlying discrepancies in the antimicrobial, antibiofilm, and antiurease activities of NPs. Such exploration would not only enhance our understanding of these variations but also enable the optimization of NP-based strategies for targeted and effective applications.

There are many advantages of using bacteria for NP production. Since NPs are used in many different industries, it is necessary to find cheaper and more efficient ways to produce them. Traditional production of various NPs chemically is costly and produces many toxic byproducts. The use of microorganisms, especially bacteria, may be a possible solution to these problems. The bacterial NP production process is cost-effective, biocompatible and non-toxic, and the process can be optimized to produce NPs with the desired properties. Therefore, it needs to be investigated using different bacteria in NP production.

Furthermore, future research should explore the long-term stability, scalability, and environmental impact of biogenic NPs to facilitate their large-scale production and practical implementation. Detailed studies on their interaction with complex biological systems, including microbiomes and host cells, are essential to predict their behavior in real-world settings. Beyond cytotoxicity, evaluating the pharmacokinetics, biodegradability, and potential for resistance development will be key to ensuring their safe and effective use. These efforts will help expand the applications of biogenic NPs not only in food, pharmaceutical, and cosmetics industries but also in agriculture, environmental remediation, and medical devices.

## Supplementary Information

Below is the link to the electronic supplementary material.

Supplementary file1 (DOCX 301 KB)

## Data Availability

The datasets generated during and/or analyzed during the current study are available from the corresponding author on reasonable request.
