# Supplementary material for: Antimicrobial, antibiofilm, and antiurease activities of green-synthesized Zn, Se, and ZnSe nanoparticles against Streptococcus salivarius and Proteus mirabilis
Source: Bioprocess Biosyst Eng. 2025 Feb 5;48(4):589–603. doi: 10.1007/s00449-025-03130-8 (PMC11928436; doi:10.1007/s00449-025-03130-8)
Supplement: Supplementary file 1 — Supplementary file1 (DOCX 301 KB) [file 449_2025_3130_MOESM1_ESM.docx]

### **Antimicrobial, Antibiofilm, and Antiurease activities of Green-synthesized Zn, Se, and ZnSe Nanoparticles against *Streptococcus salivarius* and *Proteus mirabilis***


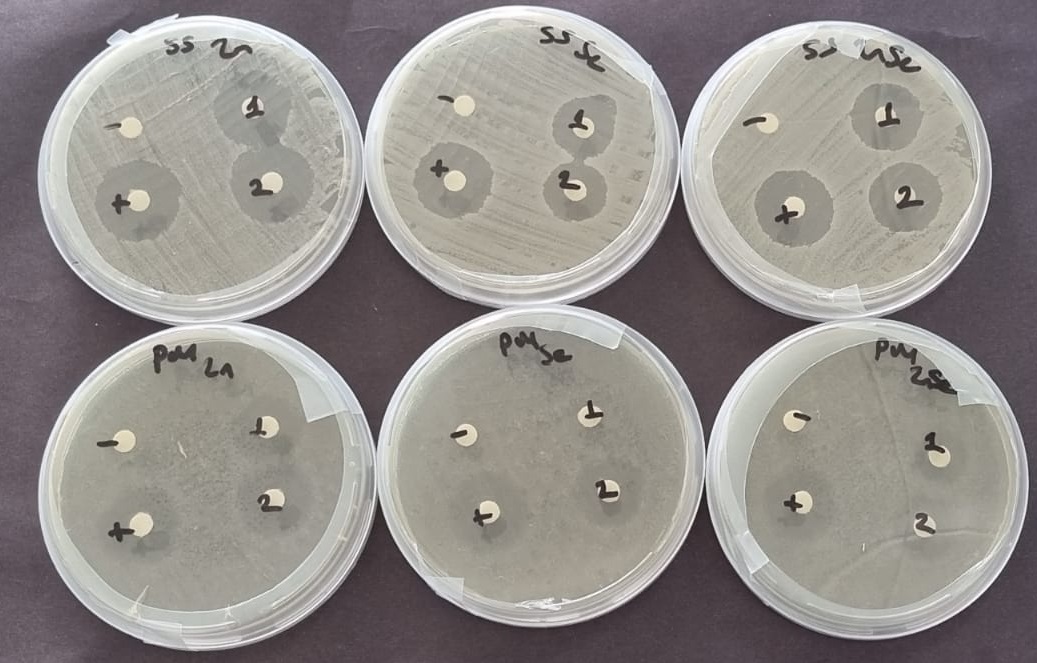


**Figure S1.** Disc diffusion tests for the evaluation of antibacterial activity of Zn, Se, and ZnSe NPs (100 and 200 μg/disc). (1) 100 μg/disc NP, (2) 200 μg/disc NP; (-) Negative control, (+) Positive control; (SS) *S. salivarius*, (Pm) *P. miribalis*


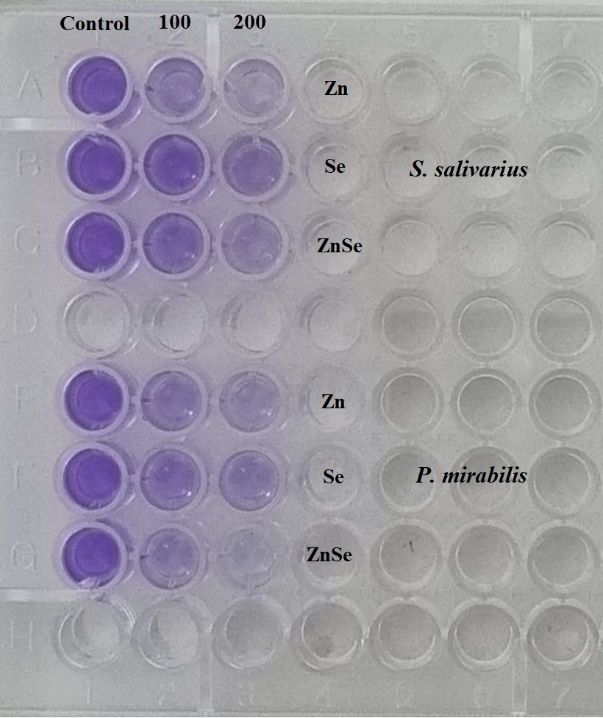


**Figure S2.** Antibiofilm activity of Zn, Se, and ZnSe NPs (100 and 200 µg/ml) on biofilm formed by *S. salivarius* and *P. miribalis*
